# Incorporating Moldable Demineralized Dentin Matrix into Treatment for a Jaw Cyst

**DOI:** 10.3390/jfb14050258

**Published:** 2023-05-07

**Authors:** Jeong-Kui Ku, Han-Wool Kwak, In-Woong Um

**Affiliations:** 1 Department of Oral and Maxillofacial Surgery, School of Dentistry and Institute of Oral Bioscience, Research Institute of Clinical Medicine of Jeonbuk National University, Biomedical Research Institute of Jeonbuk National University Hospital, Jeonbuk National University, Jeonju 54907, Republic of Korea; 2 R&D Institute, Korea Tooth Bank, Seoul 06101, Republic of Korea; h-bmp@hanmail.net

**Keywords:** bone graft surgery, demineralized dentin matrix, connective tissue graft, fistula closure, dentistry

## Abstract

The enucleation procedure is a standard treatment for jaw cysts; however, it often results in post-operative bony defects. These defects can lead to serious complications such as the risk of pathologic fracture and delayed wound healing, especially in the case of large cysts where there may be soft tissue dehiscence. Even in the case of smaller cysts, most cystic defects remain visible on postoperative radiographs and can be mistaken for cyst recurrence during follow-up periods. To avoid such complications, the use of bone graft materials should be considered. While autogenous bone is the most ideal graft material as it can be regenerated into functional bone, it has limitations due to the inevitable harvesting surgery. Many tissue engineering studies have been conducted to develop substitutes for autogenous bone. One such material is moldable-demineralized dentin matrix (M-DDM), which can aid in regeneration in cases of cystic defects. This case report highlights a patient who demonstrated the efficacy of M-DDM in bone healing for filling the cystic defect.

## 1. Introduction

Cyst enucleation is a common surgical procedure performed by oral and maxillofacial surgeons [1]. The standard protocol for treating a cyst involves complete enucleation. To achieve this, the procedure generally involves creating a bony window, which results in a larger postoperative defect than the cystic cavity. If bone grafting is not performed, spontaneous bone regeneration is unlikely to occur within six months [2]. In a recent study, Ku et al. reported a spontaneous bone healing ratio of approximately 73.5% in post-enucleation defects, as well as an altered cortical contour 12 months post-surgery, regardless of the size of the pre-operative cyst or the patient’s age [3]. This common postoperative defect may be caused by pathologic fracture or infection from food or debris [4]. A recent review article suggests that bone grafts post-enucleation accelerate the healing process, improve bone quality, and yield superior results compared to spontaneous healing [5].

Autogenous bone grafts remain the gold standard despite donor site morbidity. In 2020, Jang et al. reported that 15.3% of 216 patients who underwent autogenous bone harvest in the mandible experienced inferior alveolar nerve disturbance immediately after surgery [6]. Ku et al. suggested the grid-type cortical bone harvesting technique to prevent bone marrow invasion, which can lead to postoperative complications such as pain, swelling, and inferior alveolar nerve injury, and promote rapid recovery of masticatory function and donor site regeneration after surgery [7]. However, surgeons may hesitate to harvest the ramus due to potential donor site complications, such as morbidity, sensory disturbance, bleeding, and hematoma [8,9]. To address the continuous shortage of autogenous bone, various types of bone substitutes have been developed in the tissue engineering field. Among them, demineralized dentin matrix (DDM) has been developed from a patient’s extracted tooth and consists of hydroxyapatite, type I collagen, and matrix proteins (such as bone morphogenetic protein, fibroblast growth factor, transforming growth factors, and insulin growth factor) [10]. DDM is defined as acid-insoluble, with enlarged dentinal tubules and loosened collagen fibers due to the demineralization process. It can release the type I collagen matrix with hydroxyapatite and natural growth factors from dentin through an increased surface area resulting from the demineralization process [11]. Although the use of teeth as graft materials is initially unfamiliar to general clinicians, there is ample evidence that DDM performs well as a bone substitute and is capable of densifying its matrix and remodeling into lamellated bone histologically [6]. When bone grafting is necessary to address a large cystic defect without bone walls, maintaining the space and volume over a sustained period to prevent soft tissue ingrowth is critical for successful bone healing. Therefore, some studies suggest that the application of a resorbable or non-resorbable membrane may be necessary to provide additional stabilization of the grafted bone particles, particularly in cases of loss of periosteum or bone wall [12,13].

In 2015, Kim and colleagues introduced moldable type DDM (M-DDM; M.AutoBT, Korea Tooth Bank, Seoul, Republic of Korea) to repair sinus-related defects in implant dentistry without the use of a membrane [14]. Their findings demonstrated that M-DDM possesses remodeling capacity, as grafted DDM particles were absorbed and replaced by newly-formed bone, as confirmed by histological examination and cone-beam computed tomography (CBCT). Further research has reported the long-term outcomes of M-DDM, revealing its ability to form cortico-cancellous bone complex by CBCT that undergoes the remodeling process during functional loading [15]. In 2021, Ku and colleagues proposed that dentin-derived barriers act as osteo-inductive collagen membranes with mechanical and clot stabilities, which can replace the osteogenic function of the periosteum [16]. Given that M-DDM consists of type 1 collagen, its increased viscosity could potentially provide similar mechanical stability and block soft tissue invasion, similar to a collagen membrane. The aim of this study was to evaluate the outcomes of bone grafts using M-DDM in response to cystic defects detected with CBCT, based on the formation of the cortico-cancellous complex and cortical contour.

## 2. Case Presentation

**Figure 1:**
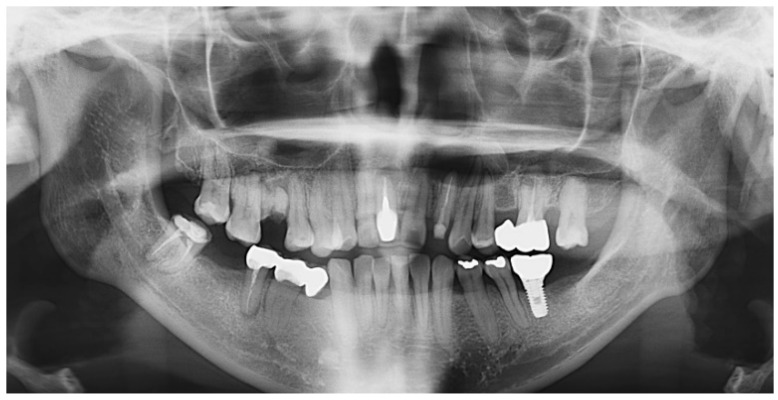
Initial panoramic X-ray. Pre-operative radiographs revealed a cystic lesion on the left anterior maxilla, and there were two teeth to be extracted: right maxillary first molar and left maxillary third molar, respectively.

A 34-year-old female patient presented with a radicular cyst on the anterior region of her left maxilla. She had no systemic disease and was a non-smoker. Radiographic imaging, including a panoramic radiograph and cone beam computed tomography (CBCT; PaX-i3D, Vatech Co., Gyeonggi-do, Republic of Korea; 0.3-mm, 24 s, 106 kV, and 65 mAs.), revealed a cystic lesion in the area of the maxillary left lateral incisor. The tooth had previously undergone endodontic treatment, but the crown had fractured. Despite the absence of tooth mobility and pain upon percussion, the patient expressed a desire to save the tooth if possible (Figure 1).

To address the issue, the patient consented to undergo bone grafting with autogenous DDM from the patient’s fractured right maxillary first molar and left maxillary third molar. These teeth were extracted during a routine procedure under local anesthesia and were sent to a manufacturer (Korea Tooth Bank, Seoul, Republic of Korea) to be processed into M-DDM. In addition, the patient was referred to an endodontist to receive re-endodontic treatment on her maxillary left lateral incisor.

**Figure 2:**
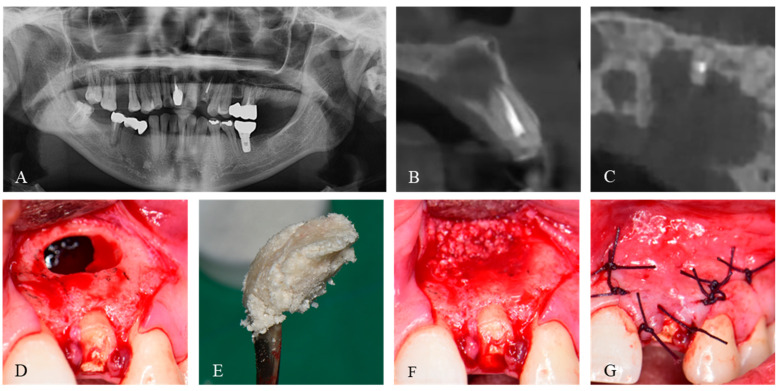
Pre- and intra-operative images. Pre-operative radiographs showed a cystic lesion on the left anterior maxilla and dehiscence of the palatal bone. (**A**) Panoramic X-ray, (**B**) Sagittal section of CBCT, (**C**) Axial section of CBCT. (**D**–**G**) Intra-operative images. (**D**) Bone defect with bucco-palatal bony dehiscence after the enucleation of cyst and apicoectomy procedure on the left maxillary lateral incisor. (**E**) After hydration of M-DDM according to the manufacturer’s instruction, M-DDM obtained a high viscosity that could mold its shape. (**F**) M-DDM was grafted into the cystic defect. (**G**) Primary closure with 4-0 vicryl.

The patient was scheduled for cyst removal and bone grafting to repair a postoperative defect once the DDM had arrived (Figure 2A). Under general anesthesia and with the infiltration of 1:100,000 2% lidocaine HCl and epinephrine (Yuhan Co., Seoul, Republic of Korea), a triangular flap was created, and the full-thickness flap was raised. The cyst removal was followed by the grinding of the buccal bone, and a horizontal apicoectomy of 3 mm from the root of the maxillary left lateral incisor was performed without retrograde preparation and filling. Palate wall loss was also observed (Figure 2B). After profuse saline irrigation and bleeding control, M-DDM was prepared. Briefly, M-DDM was hydrated with the exact amount of saline according to the manufacturer’s instruction based on the amount of DDM powder, which was fabricated with 70% demineralization of DDM and ranged size from 300–800 μm, and gently stirred for about 1 min until it reached sufficient viscosity to maintain its shape (Figure 2C). The M-DDM was then packed into the cystic defect (Figure 2D). The high viscosity of DDM particles in the M-DDM allowed their shape and position at the grafted site to be maintained, without any migration. Hence, there was no requirement to use an absorbable membrane to provide additional stability to the graft (Figure 2E). After primary closure (Figure 2F), postoperative care was prescribed as cephalosporin antibiotic (Mesexin 500 mg tid, Hanlim Pharm., Yongin, Republic of Korea) and naproxen (Naxen F 500 mg bid, Chong Kun Dang Pharm., Seoul, Republic of Korea) for 5 days.

**Figure 3:**
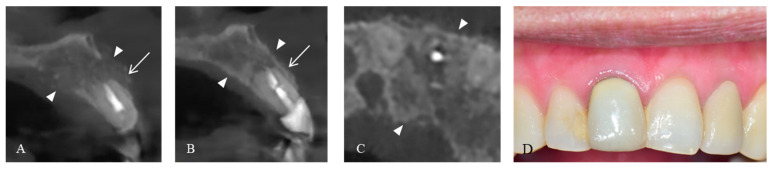
Postoperative images. (**A**) After one week, M-DDM was observed to have been successfully grafted onto the cystic defect in the area of cortical bone loss (arrowhead) and on the alveolar bone above the maxillary left lateral incisor (arrow). (**B**) M-DDM had healed into the cortico-cancellous complex, resulting in intact bucco-palatal cortical bone (arrowhead) and a significant increase in the thickness of the buccal alveolar bone around the maxillary left lateral incisor (arrow). (**C**) The axial view of CBCT also showed bone healing with cortical lining on both bucco-palatal contour (arrow). (**D**) The soft tissue around the maxillary left lateral incisor showed intact healing with sufficient volume.

The surgical wound healed without any complications, and the stitches were removed one week after the surgery. The postoperative CBCT revealed that the M-DDM was fully packed in the cystic cavity, covering the thin buccal alveolar bone of the maxillary left lateral incisor (Figure 3A). Because the patient wished to undergo dental treatment of her remaining maxillary lateral incision at a local clinic, the patient revisited the hospital for a follow-up appointment eight months after the surgery. The grafted M-DDM was remodeled into a cortico-cancellous complex without any detectable alteration in cortical contour as observed on the CBCT (Figure 3B). Furthermore, there was a significant regeneration of the buccal alveolar bone in the maxillary left lateral incisor, resulting in the formation of a cortical lining with appropriate thickness. Additionally, the gingiva adequately healed over a sufficient volume of periodontal tissue (Figure 3C).

## 3. Discussion

This study aimed to assess whether moldable demineralized dentin matrix (M-DDM) showed significant remodeling capability to transform the cortico-cancellous complex within the cystic defect. Some researchers have argued that bone grafts are unnecessary in response to cyst defects due to spontaneous healing, especially small defects less than 2 cm in size [5,17]. However, these evaluations only considered bone density as determined by plain X-ray and did not take into account any changes in contour. In 2022, a 3D based evaluation to determine the viability of spontaneous healing revealed that even a small defect (less than 3 cc) was not completely healed even after one year [3]. Consistent with a recent systematic review [5], this study suggests that bone grafting with M-DDM may offer advantages for early regeneration without shrinkage of the patient’s own contour.

Proper healing outcomes of jaw defects have been reported when the periosteum was repaired with a barrier membrane [18]. In 2014, Kitayama et al. reported that non-cross-linked collagen membrane and HA-containing collagen membrane demonstrated about 35% new bone formation when used with a biphasic scaffold, which was a superior outcome compared to using only deproteinized bovine bone [19]. In 2019, Hong et al. found that the ultraviolet cross-linked membrane showed about 36% of new bone formation in the other biphasic scaffold, which produced similar results to the chemically-cross-linked collagen membrane [20]. However, a randomized controlled trial reported that bone grafts do not contribute to increased new bone volume and density between absorbable and non-absorbable membranes [21]. Additionally, Dahlin et al. showed that bone density change of the cystic defect was not different between the group with membrane and the other group without membrane [13]. In 2015, the efficacy of M-DDM was first reported in a maxillary sinus graft surgery [14]. The viscosity of M-DDM was advantageous because it allowed the grafts to maintain their shape and location for packing under the Schneiderian membrane in the sinus cavity. There was a clinical benefit in that the high viscosity of M-DDM could be resistant even with sinus membrane perforation without an additional absorbable membrane [22]. Similar to the sinus surgery, M-DDM might provide additional benefits beyond its ability to promote bone healing in patients at high risk of palatal fistula, which could be occurred by pathologic conditions such as abscess, cysts, and tumors [23]. In this patient, M-DDM not only fill the defect but also regenerated the alveolar bone overlying the tooth (Figure 2). Kim et al. showed that DDM improved the alveolar bone healing on the exposed tooth root 4 months after the graft [24]. Their paper highlights the presence of endogenous bone morphogenic protein (BMP) in the dentin matrix, which encourages alveolar bone regeneration despite the unfavorable environment for the bone graft as the same action of BMP from bone matrix [25,26]. Regarding M-DDM, the first clinical report in 2015 showed that the remodeling capacity of M-DDM was high, as DDM particles were replaced with newly formed bone within 3–4 months, both radiographically and histologically [14]. In 2019, Ku et al. demonstrated the long-term efficacy of M-DDM in sinus graft for thin residual alveolar bone [15]. They revealed a well-formed cortico-cancellous bone complex with homogenous bone structure and maturated lamellar bone that could withstand functional loading of dental implants during a long-term remodeling process. In a histomorphometric study, Lee et al. compared the effectiveness of DDM with various other scaffolds in the sinus and observed new bone formation around the implant in all groups after 4 months [27]. Jeong et al. conducted a retrospective clinical study on 100 implants to assess the effectiveness of DDM in the sinus [28]. Histologic examination showed gradual resorption and new bone formation around the implants. In 2018, Jung et al. evaluate DDM compared with bovine xenograft and reported similar histologic and radiological aspects of newly formed bone in both grafts [29]. In 2023, Matko et al. compared DDM with a mixture of bovine xenograft with autologous bone [30]. Their immunohistochemical analysis revealed immunopositivity of TNF-α and BMP-4, indicating osteo-inductivity, and showed a similar percentage of newly formed bone between the groups.

How well a bone heals depends on the location of the defects relative to the surrounding bone and periosteum, both of which are capable of spontaneous bone repair [31]. Although there were no histological findings available for this patient postoperatively, CBCT showed that M-DDM had formed a sound cortico-cancellous complex at postoperative 8 months. The formation of this complex provides a significantly high level of clinical evidence that the graft was substituted by functional bone, consisting of cortical bone supported by cancellous bone [32]. In 2022, Wang et al. reported a systemic review for efficacy of bone grafts in jaw cystic lesion [5]. They demonstrated that the primary limitation in assessing the effectiveness of bone grafts is the variation in methods used to evaluate bone regeneration. The reduction of defect volume is considered the most reliable indicator of bone healing. However, when bone grafts are used, the volume of the defect, which is determined using CBCT or X-rays, can be affected by the density of the graft. To overcome the limitations of CBCT, Kattimani et al. proposed criteria based on changes in bone density and the contour of the bone defect [33]. However, interpreting bone density or contour could be available only after confirming that the grafted area has been remodeled into functional cortico-cancellous bone. To compare the outcome between spontaneous healing and bone grafts, as in cystic defects like the one in this patient, the time and amount of cortico-cancellous complex formation should be evaluated in CBCT, unless the grafted area reveals histologically normal bone tissue without remaining bone substitutes.

In this patient, the bucco-palatal bone wall was lost after surgery. Therefore, it was a poor condition for bone graft, similar to that of a patient with an alveolar cleft. For such cases, it is strongly recommend to apply autogenous bone or recombinant human BMP-2 (rhBMP-2) [34]. As dentin is similar in composition to bone, and has been suggested as a potential carrier of rhBMP-2 [25,29,35], the release profile of rhBMP-2 incorporated with DDM (DDM/rhBMP-2) was postulated according to the type of incorporation method [25]. There are two methods for incorporating DDM/rhBMP-2. First, the DDM scaffold can be dipped into the BMP solution and left to dry, as physical adsorption. Second, modified physical entrapment of rhBMP-2 within dentinal tubules can be achieved by freeze-drying. For clinical application, the rhBMP-2 concentration was set at 0.2 mg/mL with DDM [32,36], which is lower than the FDA-approved concentration of 1.5 mg/mL with a collagen sponge [1]. After the rapid release of incorporated rhBMP-2 in the body, dentin degradation and sequential release of BMPs in the dentin matrix occurs [25,37]. Based on a histological review [38], DDM is a suitable carrier for rhBMP-2 with continued release over 30 days at concentrations sufficient to stimulate osteogenic differentiation in vitro, while the bone formation with DDM/rhBMP-2 was greater compared with DDM alone. In addition, DDM/rhBMP-2 had time- and dose-dependent effects. These findings suggest that rhBMP-2 simultaneously initiates both osteoclastic resorption of DDM and osteo-inductive new bone formation. Through this osteoclastic resorption, promoted by rhBMP-2, enlarged dentinal tubules in the dentin matrix release several growth factors, including matrix binding and mineral binding proteins, which initiate osteoblastic bone formation to facilitate remodeling in the early stage. DDM incorporated with rhBMP-2 may be effective in such cases; for example, where there is an alveolar cleft or some jaw remains post-sequestrectomy for osteomyelitis and jaw necrosis. In particular, M-DDM incorporated with rhBMP-2 may improve bone healing by enhancing the stability of the graft. Additional studies applying diverse types of dentin grafts to a large number of patients will reveal their efficacy.

## 4. Conclusions

The viscosity of M-DDM enabled it to retain its position and shape after grafting, eliminating the need for an additional membrane. At eight months postoperatively from the cyst enucleation, M-DDM was observed to have regenerated within the cystic cavity and alveolar bone. CBCT revealed that the regenerated bone was a cortico-cancellous complex with an intact surface contour. In conclusion, M-DDM has the potential to remodel into functional cortical bone supported by cancellous bone.

## Data Availability

Not applicable.
